# Back from the Brink: The Holocene History of the Carpathian Barbel *Barbus carpathicus*

**DOI:** 10.1371/journal.pone.0082464

**Published:** 2013-12-12

**Authors:** Maciej K. Konopiński, Antoni Amirowicz, Petr Kotlík, Krzysztof Kukuła, Aneta Bylak, Ladislav Pekarik, Alena Šediva

**Affiliations:** 1 Institute of Nature Conservation, Polish Academy of Sciences, Kraków, Poland; 2 Institute of Animal Physiology and Genetics AS CR, Liběchov, Czech Republic; 3 University of Rzeszów, Department of Environmental Biology, Rzeszów, Poland; 4 Institut of Zoology, Slovak Academy of Sciences, Bratislava, Slovakia; Institute of Biochemistry and Biology, Germany

## Abstract

As a result of specific adaptations and habitat preferences strongly rheophilic fish species may show high levels of endemism. Many temperate rheophilic fish species were subjected to a series of range contractions during the Pleistocene, and then successfully expanded during the Holocene, colonising previously abandoned areas. The Carpathian barbel (*Barbus carpathicus* Kotlík, Tsigenopoulos, Ráb et Berrebi 2002) occurs in the montane streams in three basins of the main Central European rivers in the northern part of the Carpathian range. We used genetic variation within 3 mitochondrial and 9 microsatellite loci to determine a pattern of postglacial expansion in *B. carpathicus*. We found that overall genetic variation within the species is relatively low. Estimate of time to the most recent common ancestor (tMRCA) of mitochondrial sequences falls within the Holocene. The highest levels of genetic variation found in upper reaches of the Tisa river in the Danube basin suggest that glacial refugia were located in the south-eastern part of the species range. Our data suggest that the species crossed different watersheds at least six times as three genetically distinct groups (probably established in different expansion episodes) were found in northern part of the species range. Clines of genetic variation were observed in both the Danube and Vistula basins, which probably resulted from subsequent bottlenecks while colonizing successive habitats (south eastern populations) or due to the admixture of genetically diverse individuals to a previously uniform population (Vistula basin). Therefore, *B. carpathicus* underwent both demographic breakdowns and expansions during the Holocene, showing its distribution and demography are sensitive to environmental change. Our findings are important in the light of the current human-induced habitats alterations.

## Introduction

During the Pleistocene numerous temperate species were subjected to latitudinal range shifts and demographic fluctuations [1].The cooler climate and shortened vegetation period during glaciations radically altered the functioning of ecosystems. Species that were not able to establish populations in relic patches of the appropriate environment went extinct, while others that survived the colder period in glacial refugia recolonized temperate zones when the climate warmed. These range changes were determined by the ability of species to tolerate or adapt to changing conditions, and different species may thus have responded differently [2], [3]. In many species such retreat/expansion cycles were accompanied by demographic fluctuations [4].

Species dispersal depends strongly on the interconnectivity among patches of suitable environment and species with limited dispersal abilities thus may have been trapped in narrow patches of a suitable habitat. In riverine species, expansion corridors are limited to linear aquatic habitats within a river system. These pathways of expansion are isolated by watersheds, while in strongly rheophilic fishes migration may also be hindered by long stretches of lowland river segments [5]. 

The Carpathian barbel, *Barbus carpathicus* Kotlík, Tsigenopoulos, Ráb et Berrebi, 2002, was only recently described as a separate species, and knowledge of life history specific to this species is therefore scarce [5],[6],[7]. The species occurs in Northern and Central part of Carpathian Mountains. Although some local populations of *B. carpathicus* are large they are confined to montane/submontane streams and rivers [8]. 

The Carpathian barbel diverged from its closest congener Balkan barbel, *B. balcanicus* Kotlík, Tsigenopoulos, Ráb et Berrebi, 2002, in the mid-Pliocene between approximately 3.9–4.1 million years ago (mya) [6]. Both species share many biological similarities with each other and with the third rheophilic barbel occurring in the Danube basin, Peteny’s barbel, *B. petenyi* Kotlík, Tsigenopoulos, Ráb et Berrebi, 2002. The similarities are such that the three above mentioned species have been confused as a single species until recently [7], yet there is a striking difference in the level of genetic variation between *B. carpathicus* and the other two species [6]. *B. carpathicus* shows great potential as a model for analysing the natural expansion of montane fish species. It belongs to a number of southern European species of montane barbels which share some common features. They are small to medium-sized, strictly rheophilic and therefore confined to montane ranges, and almost allopatric [9]. 

A recent study carried out in a small part of this area at the Vistula–Dniester watershed [5] based on variation within the nuclear microsatellite loci found evidence that (1) the cline of microsatellite diversity decreases eastwards, (2) the overall low genetic variation reflects probable population bottlenecks, and (3) that there are genetic similarities between populations in the adjacent rivers in the basins of the Vistula and Dniester. Such features of the populations of *B. carpathicus* north of the Carpathians resemble the gradual loss of genetic diversity which accompanies many postglacial expansions of species limited by hard barriers that permit only rare migrations [10], [11], [12], [13]. 

The aim of this study was to determine the pattern of expansion of *B. carpathicus*, i.e. (1) where the Pleistocene refugia were located, (2) whether *B. carpathicus* invaded rivers of the Baltic catchment’s northern slopes of the Carpathians on a single or multiple occasions, (3) where the most likely points are at which the main Carpathian watershed was crossed, including whether the founders originated from a single or multiple sources, (4) what the likely origin of clines of genetic variation is, and (5) at what time these expansion events occurred, in particular whether the Vistulan populations were established in the Holocene or contain traces of earlier expansions or presence of glacial refugia. We addressed above question using sequence variation within mitochondrial haplotypes and length variation in nine nuclear microsatellite loci.

## Materials and Methods

### Ethics statement

Standard procedures used in aquaculture for catching and marking fish were employed in the course of the study. No in vivo experiments were performed on animals. Permissions for catching and fin clipping were granted by the Voivode of the Małopolska region, Poland, Nos. RG.I.6052-1/09, RG.I.6052-2/09, and RG.I.6052-6/09, Voivode of the Podkarpacie Province, Poland, Nos. RŚ.I.EK.6251/03/09 and RŚ.I.6251/08/08, Voivode of the Silesia Province, Poland, No: 3905/TW/2009, Ministry of Environment of the Slovak Republic: Nos. 47/2008, 48/2008, 18/2009, 19/2009, and 6057/2010-2.1. 

No animals were sacrificed in the course of this study. The muscle samples were from previous published studies [6], [7] and were collected in 1997-1998. The sample collection was overseen by the Ethics Committee of the Institute of Animal Physiology and Genetics, Academy of Sciences of the Czech Republic. Fish were sacrificed using an overdose of anesthetics and muscle samples were taken from dead fish.

### Sample collection

**Figure 1:**
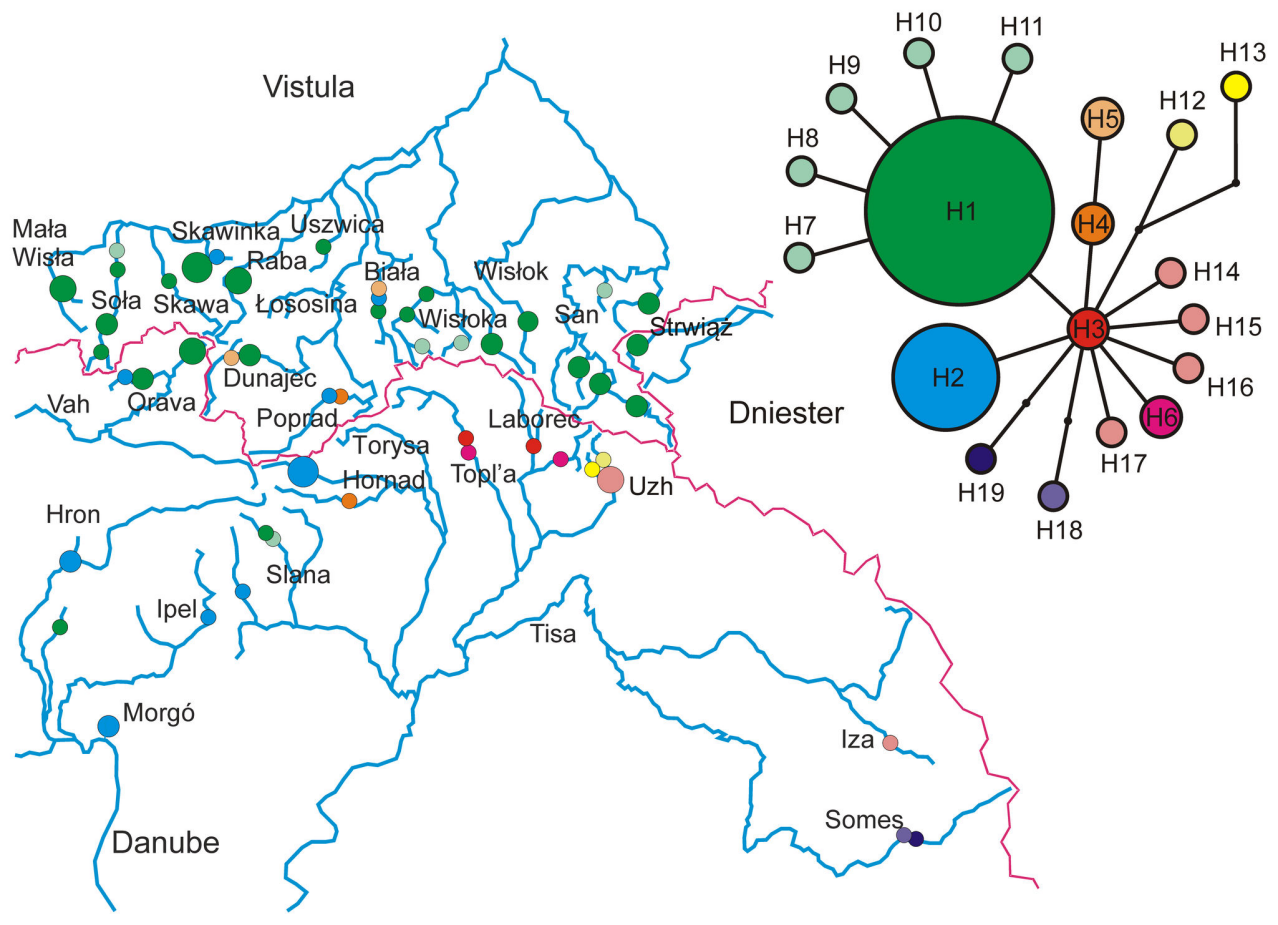
Statistical parsimony network of mitochondrial haplotypes, and the distribution of haplotypes in current range of *Barbus carpathicus*.

A total of 841 samples from 27 populations were analyzed. Sampling covered rivers from all three basins within the species range (Table 1, Figure 1), i.e. the Danube, Vistula and Dniester. To avoid analyzing single flocks, samples from several locations along the river were collected. Samples were pooled into distinct populations when sampling locations were divided by long segments of lowland river or the distance between sampling locations exceeded 100 km (e.g. Poprad vs. Dunajec). A unique sample collected in the Sikenica river belonging to the Hron basin was included in the Hron sample. 

**Table 1:** Characterization of polimorphic microsatellite loci successfully amplified in the Carpathian barbel.

Primer set	Fluorescent dye	Annealing temp.	Locus	Product size (bp)	*N* alleles	*H_E_*
Barb 37	FAM-6	56	Barb 37	201-224	10	0.43
Barb 59	TAMRA	56	Barb 59	134-258	41	0.87
Barb 79	FAM-6	54	Barb 79	200-216	5	0.53
LC 293*	Hex	56	LC 293b	100-110	5	0.50
LCo 4*	Hex	56	LCo 4a	207-211	5	0.13
			LCo 4b	212-214	3	0.47
MFW 17*	FAM-6	54	MFW 17a	180-190	3	0.14
			MFW 17b	199-221	9	0.50
MFW 19	TAMRA	54	MFW 19	187-297	22	0.80

^*^ primers amplifying duplicated loci.

The majority of samples (625) were fin clips collected in the years 2008–2009. Fish were immobilized with a portable pulsed-DC electroshocker, and after clipping 5–10 mm^2^ of the tip of the pelvic fin they were released in the place of capture. Samples were preserved in 96% ethanol and stored in −20 °C upon arrival to the laboratory. 

We also used 66 fin clips collected for a previous study on the expansion of *B. carpathicus* [5]. To obtain muscle samples fish were euthanized using an overdose of 2-phenoxyethanol (bath in 0.4 mg/liter solution) without any prior manipulation. 

### Laboratory protocols

Immediately prior to DNA isolation, samples were desiccated overnight at 60 °C. Genomic DNA was isolated with NucleoSpin Tissue Kit (Macherey & Nagel), following the standard manufacturer’s protocol. 

Three fragments of mitochondrial genome were amplified and sequenced in 78 individuals of *B. carpathicus* including the cytochrome *b* (*cytB*, 1193 bp): L14724 and H15915 [14], NADH dehydrogenase subunit 2 (*ND2*, 1142 bp): ND2-2F (5’-CTTCCTTTACACCACTTTCT-3’) and ND2-1R (5’TTGAAGGCTTTTGGTCTAAT-3’), ATPase subunits 6 and 8 (*ATP*, 809 bp): ATP8.2 and Co3.2 [15]. Both PCR conditions and thermal profiles were the same for the three loci. PCR reactions were prepared in 20 µl volumes containing 20 ng of template DNA, 200 nM of each primer (Genomed), 2 mM MgCl_2_, 200 nM dNTP, 1 x PCR buffer containing NH_4_SO_4_ and 1 U Taq DNA polymerase (Fermentas). Loci were amplified in PTC-200 MJResearch Thermal Cycler under the following thermal profile: 3 min initial denaturation at 94 °C, 10 cycles of 30 s at 94 °C, 45 s at 58 °C decreasing by 0.5 °C each cycle, 2 min at 72 °C, and 22 cycles of 30 s at 94 °C, 45 s at 52 °C, 2 min at 72 °C. After the PCR, the products were purified using modified Exo-SAP method [16] with 10 U of Exonuclease I (Fermentas) and 0.5 U of FastAP (Fermenats). The PCR products were sequenced with the primers used in amplification reactions and with the following internal primers – *cytB*: CytB-IntF [17] and CytB-Int2R (5’-ATTTGACCCTGTTTCGTGGA-3’), *ND2*: ND2-IntF (5’-ATTCAAACAGCACAAACCAT-3’) and ND2-IntR (5’-TCGTAGTTGGGTTTGATTTA-3’). Sequencing reactions were performed using BigDye3.1 chemistry (Applera). Sequencing products were sequenced with AbiPrism 3130xl Genetic Analyser (Applera). Chromatograms were preanalyzed with Sequencing Analysis Software ver. 5.3 and assembled in contigs using SeqMan program from DNAStar package (Lasergene). Sequences were aligned and trimmed to the shortest available sequence using Bioedit ver. 7.1.11 [18]. Due to technical problems not related to DNA polymorphism, we only obtained sequences from the 544 bp fragment of *ND2* gene. For the *ATP* locus we obtained a fragment of 842 bp in all sequences while in the *cytB* gene the entire amplified fragment of 1193 bp was successfully sequenced. All the unique haplotypes have been submitted to the NCBI GenBank nucleotide database under following accession numbers: KF819397-KF819405 and KF826494-KF826507. 

Previous studies demonstrated that *B. carpathicus* possess relatively low genetic variation as compared to its congeners [5], [6], [7]. Only seven primer pairs out of forty-five tested amplified polymorphic microsatellite loci in the Carpathian barbel: Barb37, Barb59, Barb79 [19], MFW17, MFW19, [20], Lco4 [21], LC293 [22] (Table 1). Loci were amplified in MJResearch PTC-200 Thermal Cycler in 10 µl reactions containing 1x HotStar Master Mix Kit (Qiagen), 200 nM of each primer, and 20 ng of template DNA, using the following thermal profile: denaturation/enzyme activation – 15 min at 95 °C; 32 cycles of: 30 s at 94°C, 40 s at the locus optimal annealing temperature (Table 1), 50 s at 72 °C; final extension – 5 min at 72°C. The efficacy of amplification in varying PCR conditions was validated on 3 DNAs of *B. carpathicus* from the Somes, Béla Orava and Dunajec rivers. Product lengths were estimated in GenMapper 4.0 (Applera) based on chromatograms from AbiPrism 3130xl Genetic Analyser (Applera) by comparison to Rox-400HD size standard (Applera). 

### Statistical analyses

#### Genetic variation within populations

Levels of genetic variation were estimated overall and within populations using both mitochondrial sequences and microsatellite allele frequencies. Because the sequence variation in mitochondrial genome was low, all three regions were concatenated. For the 2,579 bp fragment of mtDNA basic statistics such as number of haplotypes (*h*), number of segregating sites (*S*), nucleotide and haplotype diversities (π and *H*_*d*_) were calculated in DnaSP ver. 5.10.01 [23]. The evolutionary relationships between haplotypes were estimated by maximum parsimony using TCS ver. 1.21 [24]. 

The expected heterozygosity (*H*_*E*_) and Shannon index of diversity (SI) were calculated from microsatellite data with MSA ver. 4.05 [25], whereas allelic richness (*R*_*S*_) and inbreeding coefficient (*F*_*IS*_) were calculated in Fstat ver. 2.9.3.2 [26]. The Ipel river population, represented by only 2 individuals, was not included in *R*_*S*_ calculation. Mean values across all loci within each population were calculated. Because the location of the amplified loci is unknown we have tested the genotype frequencies for signs of linkage disequilibrium using Fstat. In each population we performed locus by locus exact test of Hardy-Weinberg equilibrium as implemented in Arlequin ver. 3.5.1.3 [27] with 1,000,000 steps in Markov chain and 100,000 dememorization steps.

#### Demographic history of the species

To check for a sign of recent population expansion in the variation of mtDNA sequences, Tajima’s *D* and Fu’s *F*_*S*_ were calculated in Arlequin and tested for significance using 10,000 simulations. The pattern of expansions was further tested based on the distribution of pairwise differences between haplotypes [28]. Mismatch distributions were analyzed in Arlequin using both demographic and spatial expansion models [29]. The statistical support of the two models was tested using the sum of squared deviations (SSD) with 10,000 bootstrap replicates. 

Time to the most recent common ancestor (tMRCA) of the mitochondrial genome was estimated by means of Bayesian inference as implemented in Beast ver. 1.7.5 [30]. The Bayesian Skyline coalescent tree prior was selected as the most likely demographic scenario for the species – results of other tests (Tajima’s *D*, Fu’s *F*_*S*_, mismatch distribution, see the Results) suggest that the species has undergone expansion since the last glacial period, so constant population size in this period is very unlikely. On the other hand, clear limits for further expansion occur in the study area, thus simple expansion tree priors would poorly reflect the actual demographic history of the species. Because there are no long branches in the haplotype tree a simple skyline model with constant population size between coalescent points was used. First, short runs of fifty million generations sampled every 1000 generations were performed to choose the most likely molecular clock model and sequence substitution models. The resulting log files were inspected in Tracer ver. 1.5 (Rambaut and Drummond, http://tree.bio.ed.ac.uk/software/tracer/). Twenty million generations at the beginning of each run were trimmed as the burn-in period after which chains reached stationarity. Based on the analysis of Bayesian factors, the exponential relaxed clock model was selected while no important differences were detected between different substitution schemes, thus the HKY model without data partitioning and site heterogeneity was selected as the simplest model available in the program. After completing preliminary runs, 5 runs of 500,000,000 generations sampled every 10 000 generations were performed using the selected model. The chains, after trimming the burn-in period required to reach stationarity, were combined in LogCombiner, ver. 1.7.5. Time estimates were obtained using the mean (0.07 changes/site/Myr) and standard deviation (0.032) calculated from mutation rate estimates from recent geological events (isolation events 6–9 from Table 1 in [31]). A Bayesian Skyline Plot was constructed to show the demographic history of the species and the tree root height was used as an approximation of tMRCA of the whole mitochondrial variation. 

#### Genetic structure

For all pairs of populations *F*_*ST*_ [32] was calculated in MSA ver. 4.05. Statistical support was estimated based on 10,000 permutations. Factorial correspondence analysis (FCA) was performed in Genetix ver 4.05.2 [33]. 

An assignment test was performed in Structure ver. 2.3.4 [34]. Large pairwise *F*_*ST*_ values between populations from distinct river basins (see the Results) suggest that current levels of gene flow are relatively small, thus, simpler, less parameterised model without admixture was selected. The model is appropriate to represent populations between which rates of gene flow are low and is more powerful at detecting subtle structure [34]. Initial runs of the program showed that clustering is very poor due to the low level of variation in the analyzed loci, therefore, LOCPRIOR was used to assist clustering [35]. Correlated allele frequencies between populations were assumed to minimize spurious clustering between populations. Markov Chain Monte Carlo simulations were run for 10^6^ generations with an initial burn-in period of 10^5^ generations. Runs were repeated 10 times for K ranging from 1 to 10. The method of Evanno [36] was used to detect the most probable number of clusters as implemented in the StructureHarvester website [37]. For selected simulations the cluster membership probabilities were estimated in Clumpp [38]. 

Although, Hubisz et al [35] demonstrated that using LOCPRIOR does not lead to discovery of false population structuring, robustness of the assignment was confirmed in TESS ver. 2.3.1 [39]. The Bayesian clustering algorithm implemented in TESS incorporates geographical location as an additional prior [40]. The analyses were repeated 10 times for K=2 and 3, using model without admixture. The algorithm was run for 100,000 sweeps with 10,000 sweeps as the burn-in period. 

## Results

### Mitochondrial DNA

Within the sequenced 2,579 bp of mitochondrial DNA (~15 % of a complete mitochondrial genome [41]) analysed in 78 individuals we found 19 haplotypes (Figure 1) and 22 polymorphic sites. The largest pairwise difference found between haplotypes was 5 mutations (0.2 % divergence). The mean time to tMRCA of all the haplotypes of *B. carpathicus* was estimated by Beast to correspond to 7.8 thousand years BP (95% HPD: 19.3–1.15 kya). The populations from the three major basins were not reciprocally monophyletic (Figure 1). The overall most frequent haplotype was H1, and although it was found in all 3 river basins, H1 (and its mutational variants) clearly predominated in the river basins of the Vistula tributaries. The second most abundant haplotype H2 was most frequent in the Danube basin, except in the tributaries of the Upper Tisza river (the Topl’a, Laborec, Uzh, Iza and Somes), where the populations carried H3 and its derivates. 

### Microsatellite loci

Out of 45 primer pairs tested, only 7 pairs amplified polymorphic loci in *B. carpathicus* (Table 1). Primers MFW17 and Lco4 amplified duplicated (encoded as “a” and “b”), polymorphic loci scorable in *B. carpathicus*, while primers LC293 probably amplified duplicated loci with only one locus polymorphic – the allele in the monomorphic locus possess a similar stutter band pattern as the alleles in the polymorphic locus. The number of alleles found in *B. carpathicus* varied between 3 (Lco4b) and 41 alleles (Barb59) per locus. Allelic richness (*R*_*S*_) and expected heterozygosity (*H*_*E*_) were lowest in Lco4a (1.6 and 0.13 respectively) and highest in Barb59 (6.4 and 0.87 respectively). Genotype frequencies neither show signs of linkage disequilibrium nor deviate significantly from Hardy-Weinberg expectations, except for the population sample from the Hron river basin (including sample from the Sikenica river) that showed strong heterozygote deficiency (*F*_*IS*_ = 0.406, Table S1). 

### Genetic variation within populations

**Figure 2:**
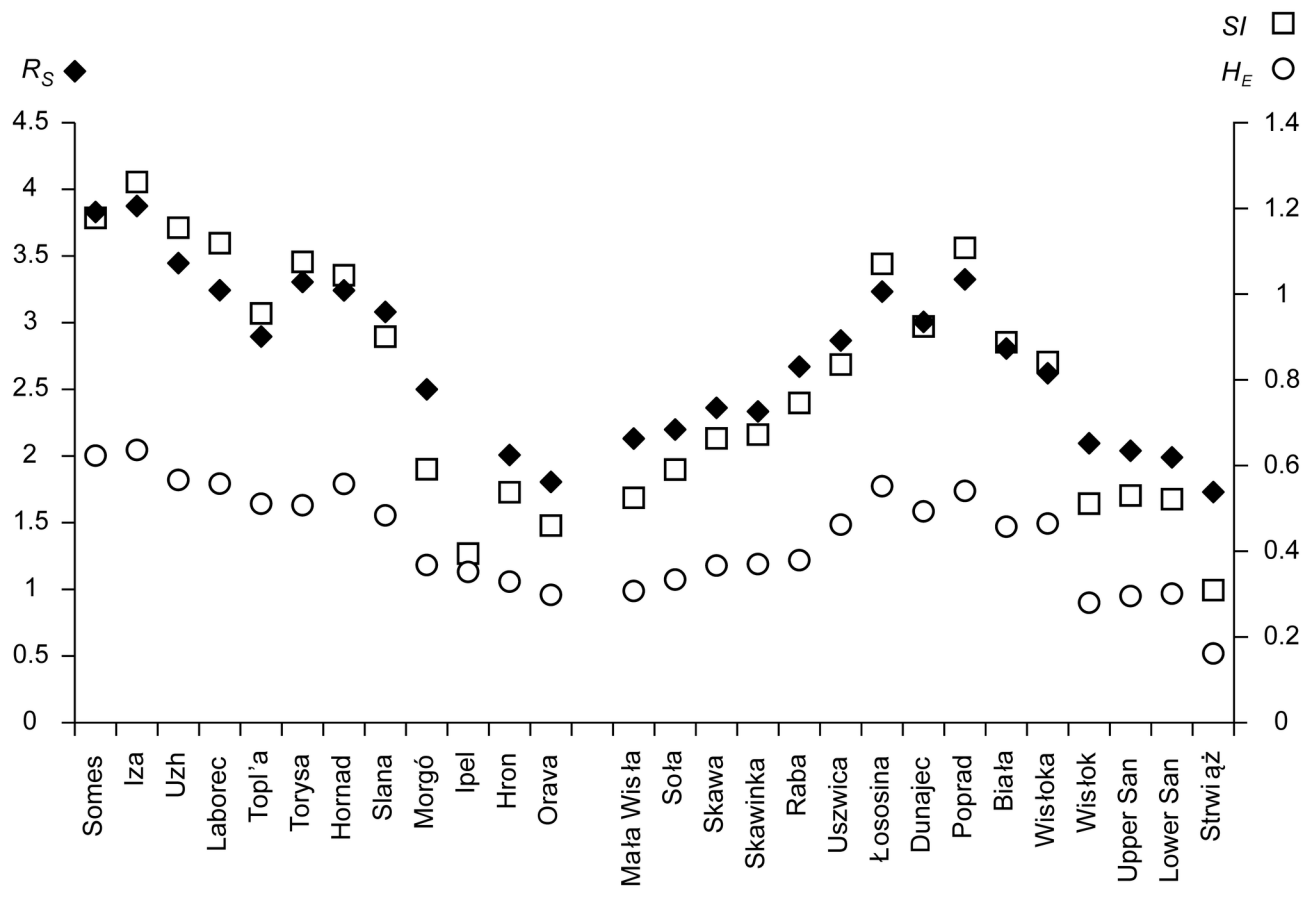
Levels of genetic variation in the 27 populations of *Barbus carpathicus*.

There were substantial differences in genetic variation levels among the populations studied (Table 2, Figure 2). Both in the mitochondrial sequences and in the microsatellite allelic variation the populations from the Danube basin were more variable (*h* = 14, *S* = 17, π = 7.3 *10^-3^, *H*_*E*_ = 0.48, *R*_*S*_ = 3.02) than those from the Vistula and its tributaries (*h* = 8, *S* = 8, π = 3.1 *10^-3^, *H*_*E*_ = 0.40, *R*_*S*_ = 2.55). The highest number of private haplotypes (8) was found in the Tisa basin, while in the Vistula there were 5 private haplotypes. No private haplotypes were found in the Strwiąż stream (the Dniester basin), however, only 2 haplotypes from that population were obtained. 

**Table 2:** Levels of genetic variation in 2,579 bp region of mitochondrial DNA and nine nuclear microsatellites across 27 populations of *Barbus carpathicus*.

**No.**	**Population**	**N_mt_**	***H_d_***	***π***	**N_MS_**	***H*_*E*_ ± S.D.**	***R*_*S*_ ± S.D.**	***SI* ± S.D.**
1	Somes	2	1	0.00155	11	0.62 ± 0.186	3.83 ± 1.96	1.18 ± 0.60
2	Iza	1	-	-	8	0.64 ± 0.214	3.87 ± 2.48	1.26 ± 0.76
3	Uh	5	1	0.00116	27	0.57 ± 0.250	3.45 ± 2.21	1.15 ± 0.79
4	Laborec	2	1	0.00039	36	0.56 ± 0.245	3.24 ± 1.98	1.12 ± 0.75
5	Topl’a	2	1	0.00039	25	0.51 ± 0.244	2.90 ± 1.90	0.95 ± 0.69
6	Torysa	-	-	-	42	0.51 ± 0.281	3.31 ± 2.22	1.08 ± 0.85
7	Hornad	6	0.6	0.00034	18	0.56 ± 0.196	3.24 ± 1.70	1.04 ± 0.60
8	Slana	3	1	0.00078	14	0.48 ± 0.258	3.08 ± 1.91	0.90 ± 0.67
9	Morgó	2	0	0	5	0.37 ± 0.361	2.50 ± 2.00	0.59 ± 0.63
10	Ipel	1	-	-	2	0.35 ± 0.348	N/A	0.39 ± 0.40
11	Hron	3	0.67	0.00052	10	0.33 ± 0.299	2.01 ± 1.27	0.54 ± 0.54
12	Orava drainage	6	0.33	0.00026	18	0.30 ± 0.233	1.81 ± 0.76	0.46 ± 0.37
	**Danube basin**	**33**	**0.84**	**0.00073**	**216**	**0.48 ± 0.117**	**3.02 ± 0.67**	**0.89 ± 0.31**
13	Mała Wisła	3	0	0	30	0.31 ± 0.267	2.13 ± 0.89	0.52 ± 0.47
14	Soła	5	0.4	0.00016	48	0.33 ± 0.293	2.20 ± 1.16	0.59 ± 0.57
15	Skawa	1	-	-	24	0.37 ± 0.262	2.36 ± 1.17	0.66 ± 0.51
16	Skawinka	5	0.4	0.00031	28	0.37 ± 0.256	2.33 ± 1.12	0.67 ± 0.50
17	Raba	3	0	0	30	0.38 ± 0.296	2.67 ± 1.65	0.75 ± 0.67
18	Uszwica	1	-	-	12	0.46 ± 0.284	2.87 ± 1.89	0.84 ± 0.65
19	Łososina	-	-	-	31	0.55 ± 0.206	3.23 ± 1.75	1.07 ± 0.65
20	Dunajec	3	0.67	0.00078	19	0.49 ± 0.231	3.01 ± 1.78	0.92 ± 0.65
21	Poprad	2	1	0.00078	54	0.54 ± 0.235	3.33 ± 1.99	1.11 ± 0.78
22	Biała	3	1	0.00103	68	0.46 ± 0.234	2.81 ± 1.58	0.89 ± 0.64
23	Wisłoka	6	0.6	0.00026	76	0.46 ± 0.241	2.62 ± 1.41	0.84 ± 0.57
24	Wisłok	2	0	0	42	0.28 ± 0.294	2.10 ± 1.30	0.51 ± 0.58
25	Lower San	9	0.22	0.00009	61	0.30 ± 0.298	2.04 ± 1.16	0.53 ± 0.57
26	Upper San				70	0.30 ± 0.281	1.99 ± 1.07	0.52 ± 0.52
	**Vistula basin**	**43**	**0.41**	**0.00031**	**593**	**0.40 ± 0.093**	**2.55 ± 0.45**	**0.74 ± 0.20**
27	Strwiąż (Dniester basin)	2	0	0	32	0.16 ± 0.219	1.73 0.96	0.31 ± 0.43

N_mt_ – number of haplotypes analysed, N_MS_ – number of individuals typed at microsatellite loci. Further explanations in text.

The highest level of variation among all populations was found in south-eastern part of the species range (the Somes and Iza rivers), while the lowest values characterised the populations from the Strwiąż (the Dniester river basin). A very low level of variation was also observed in the San, Mała Wisła, and Orava basins, clines of variation could be observed across the tributaries of the Tisa/Danube with the levels of variation decreasing westwards, and in the Vistula river system with the variation decreasing both east- and westwards and with the populations from the Dunajec basin being most variable. 

### Genetic structure

**Figure 3:**
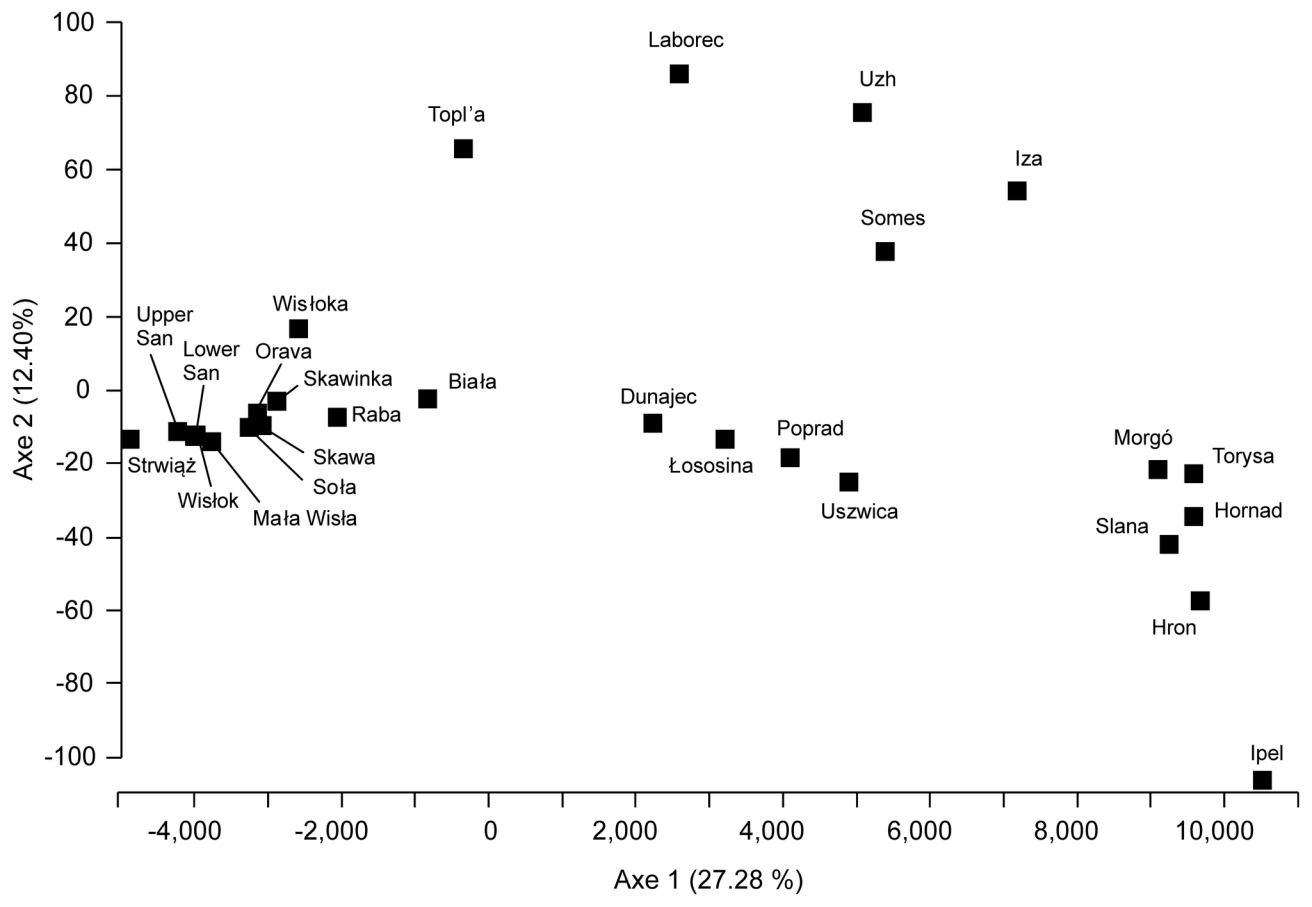
Results of factorial correspondence analysis (FCA) performed in Genetix, showing multivariate relationships among populations using microsatellite variation.

Despite the low levels of variation, the species shows surprisingly strong genetic structuring. In 351 pairwise *F*_*ST*_ estimates only 39 values (11.1%) did not differ significantly from 0, while 125 (35.6 %) were higher than *F*_*ST*_ = 0.2 (Table S1). The highest value was found between the populations in the Strwiąż and Ipel (*F*_*ST*_ = 0.717) and the lowest was between populations in the Łososina and Dunajec (−0.005). In general, *F*_*ST*_ values among populations from the Vistula, Danube, and Dniester river basins were higher than among population within those basins, but with several notable exceptions. The estimated *F*_*ST*_ between the Orava drainage and remaining populations within the Danube basin were higher (mean *F*_*ST*_ = 0.311 ±0.125) than with populations from the other two basins (mean *F*_*ST*_ = 0.157±0.0.56), and the lowest *F*_*ST*_ among the Orava basin and other populations was found in comparison with the Mała Wisła, Soła, Skawa, Skawinka, and with the Wisłoka rivers. The lowest *F*_*ST*_ values found between rivers of the Vistula basin and rivers from other basins were between the pairs Poprad–Hornad (*F*_*ST*_ = 0.051), Łososina–Uh (*F*_*ST*_ = 0.055), and Łososina–Somes (*F*_*ST*_ = 0.063), and these values were lower than in comparison with other rivers from the Vistula basin other than those of the Dunajec river system and the Uszwica river. The same pattern can be observed in factorial correspondence analysis (Fig. 3). The first axis explaining 27.28 % of variation, places south-eastern populations and populations from the Dunajec, Łososina, Poprad and Uszwica rivers in the centre, remaining Vistulan populations and the Orava basin on one side and populations from the south-western part of the range on the opposite side. The second axis groups south-eastern populations at the top of graph and the remaining ones at the bottom. 

**Figure 4:**
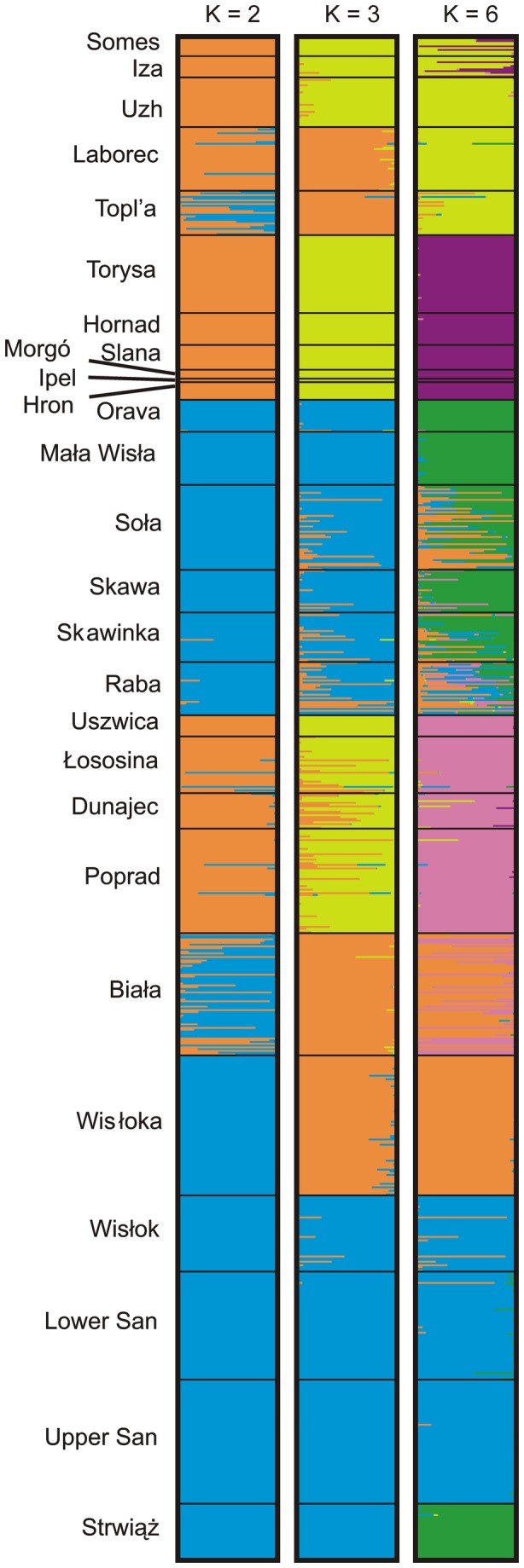
Proportional membership (*q*) of all individuals genotyped at microsatellite loci, for 3 different number of clusters (K = 2, 3, 6) identified by Structure.

An analysis of *K* based on runs of Structure indicated that the most likely number of clusters is *K* = 2 (*K* = 314), but the results for *K* = 3 and *K* = 6 are also presented as their *K* was notably higher (8.0 and 5.7 respectively) than for other *K*s (*K* < 1). For *K* = 2, most samples from the Danube basin strongly assign to a single cluster (O_*2*_), except for the Orava basin assigned to the second cluster (B_*2*_), and several individuals from populations in the Topl’a and Laborec rivers with uncertain clustering (Fig. 4). In the Vistula basin, populations from the Dunajec, Poprad, Łososina, and Uszwica rivers were assigned to the *O*_*2*_ cluster while populations from the Mała Wisła, Soła, Skawa, Skawinka, Raba, Wisłoka, Wisłok, San rivers along with population of the Strwiąż river from the Dniester basin to the *B*_*2*_ cluster. The assignment of the individuals from the Biała river was uncertain. When *K* = 3 was assumed, a major cluster from the Danube basin (Y_*3*_) was found in most populations except for the Orava basin, Topl’a, and Laborec rivers, which also contained individuals from the Dunajec, Poprad, and Uszwica rivers. Individuals from the Topl’a and Laborec rivers were assigned to the same cluster (O_*3*_) as from the Wisłoka and Biała rivers, while the third cluster (B_*3*_) unambiguously gathers population from the Orava basin (the Danube basin), Mała Wisła, Wisłok, San and Strwiąż rivers (the Dniester basin). Populations from the Raba, Skawinka, Skawa and Soła rivers were clustered either to *O*_*3*_ or *B*_*3*_. In the third test with *K* = 6, the only cluster divided between river basins was cluster *G*_*6*_ found in all 3 river basins (the Orava drainage, the Mała Wisła and Strwiąż rivers). The remaining Danubian populations were assigned strongly to either of two clusters (*Y*_*6*_ and *V*_*6*_). In the Vistula basin the Dunajec, Poprad, Łososina and Uszwica populations formed cluster *P*_*6*_, populations from the Wisłok and San rivers were grouped into cluster *B*_*6*_, all individuals from the Wisłoka river were assigned to clade *O*_*6*_, and individuals from other populations were assigned to two (Biała – P_*6*_+B_*6*_) or more clusters (Raba – G_*6*_+P_*6*_+B_*6*_+O_*6*_, Skawinka and Soła – G_*6*_+O_*6*_+B_*6*_, Skawa – G_*6*_+O_*6*_+P_*6*_).

**Figure 5:**
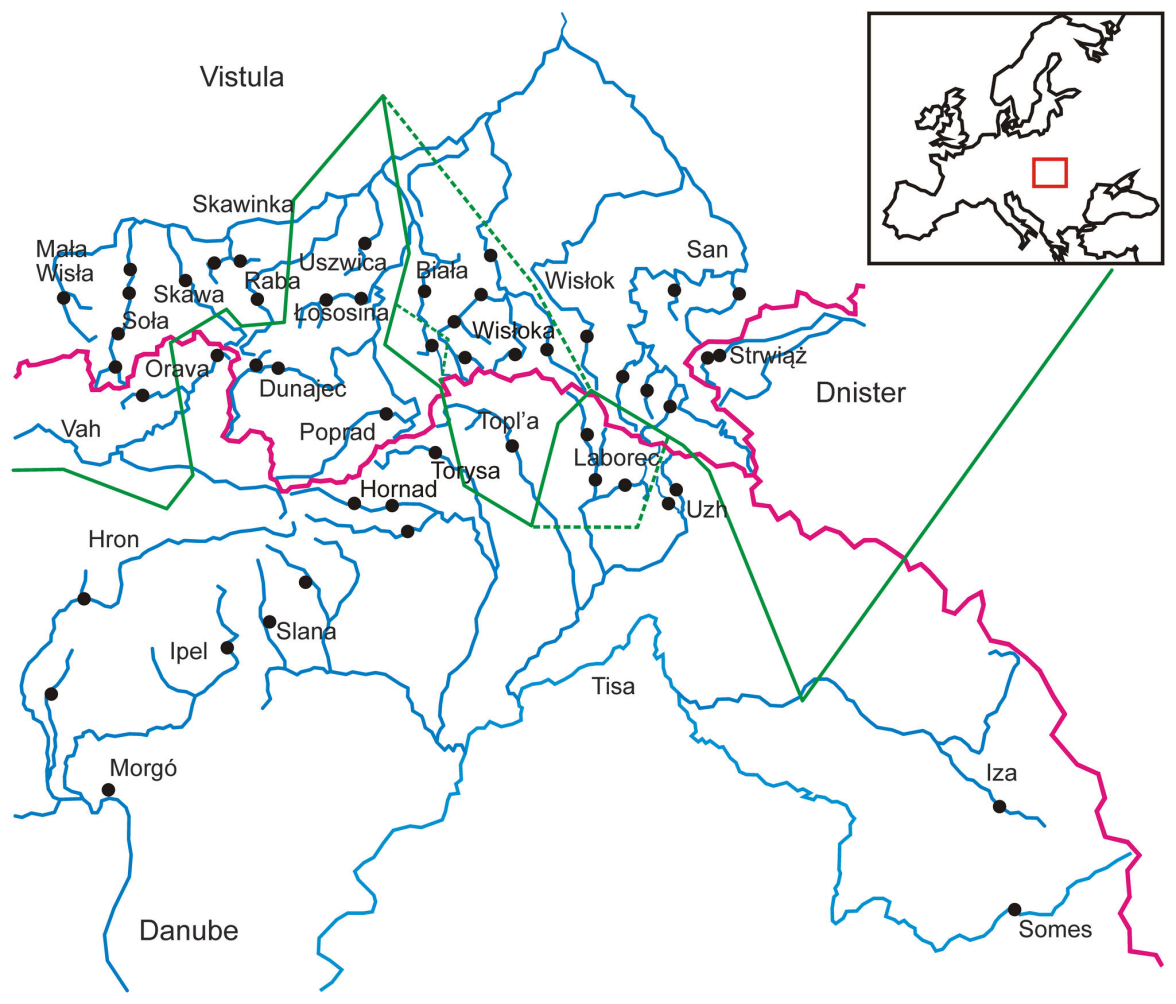
Spatial distribution of clusters detected in an assignment test performed in TESS. Solid green line – border between clusters when K = 2, dashed line – borders of the 3^rd^ cluster for K=3.

Analyses in the TESS program were performed for the most likely K = 2, and K = 3. For K = 2, populations from the Danube river basin were clustered together except for one population in the Orava basin that was clustered with most populations from northern slopes of the Carpathians except for populations in the Uszwica, Łososina, Dunajec and Poprad rivers (Figure 5). For K = 3, an additional cluster included populations from the Laborec, Topl’a in the Danube basin and the Wisłoka, and one population in the Biała in the Vistula basin.

### Historical demography

**Figure 6:**
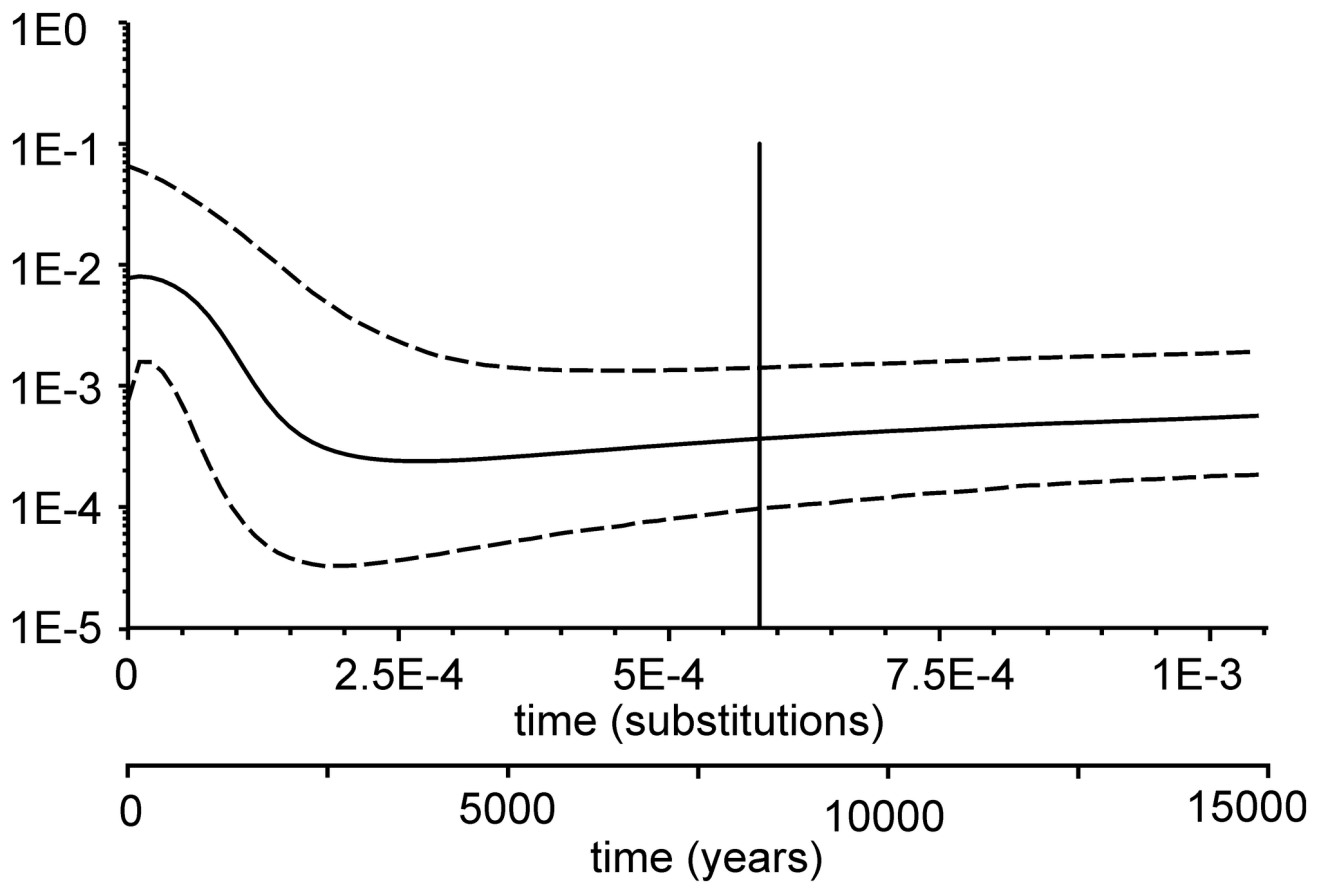
Bayesian Skyline Plot of population dynamics of the Carpathian barbel. Thick curves represent mean inferred effective population size, dashed curves mark the 95% highest probability density (HPD) intervals. Vertical line – mean mitochondrial tMRCA estimate of the whole species.

Signs of population expansion were inferred from the majority of the tests (Table 3, Figure 6). Tajima’s *D* and Fu’s *Fs* have statistically significant negative values both in the whole species and in the two major river systems analysed separately – the Danube and the Vistula. Expansion patterns tested with mismatch distribution were different depending on the population studied. In the whole species, the mismatch distribution analysis did not yield statistically significant results. The population in the Danube basin showed moderate signs of both demographic and spatial expansion (p = 0.01), while the population from the Vistula basin showed strong evidence for demographic expansion (p < 10^-5^) while analysis of spatial expansion was not statistically supported (p = 0.72). The Bayesian Skyline Plot also shows a strong expansion trend since approximately 4000 years BP when the whole species appears to have reached its lowest effective population size (Figure 6). The expansion trend stabilizes a few hundred years BP, with a slightly negative tendency at the beginning of the coalescent simulation. 

**Table 3:** Results of Tajima’s *D*, Fu’s *F*_*S*_ and mismatch distribution tests of *Barbus carpathicus* expansion along with their statistical support.

Population	Tajima		Fu		Demographic expansion model		Spatial expansion model	
	*D*	p	*F_S_*	p	SSD	p	SSD	p
Whole range	-2.05	0.002	-14.279	<10^-5^	0.051	0.114	0.036	0.226
Danube	-1.84	0.012	-8.31	<10^-5^	0.065	0.01	0.064	0.01
Vistula	-1.61	0.036	-4.31	0.006	0.224	<10^-5^	0.001	0.72

Despite using various model settings, all attempts to run the coalescent simulations for smaller subsets of sequences failed. Probably due to low variation, the chains generated in these runs mixed poorly and some of the parameters were trapped in local peaks of probability or optimization tended towards values below or above the limits of variables handled by the program. 

## Discussion

### Glacial refugia and colonization routes

All the variation presently found in the Carpathian barbel apparently was accumulated only since the last glacial maximum (LGM, ~22 kya). Most variation that could have accumulated in the species since its divergence from other species was thus erased during the late Pleistocene/early Holocene which invokes some dramatic demographic collapse across all the species range. The species probably survived the Pleistocene in glacial refugium located in the Upper Tisza river system [7] which is supported by the highest levels of genetic variation found in populations in the northern Transylvania. Mean tMRCA estimated with an upper bound located right after the LGM would appear to suggest that the Holocene was a turning point in the recent history of *B. carpathicus*. Due to a complete lack of variation from the earlier history of the species, we cannot answer if this is a result of a single demographic collapse event or an effect of prolonged genetic drift during the Weichsel glaciation (110–10 kya). Because long stretches of lowland rivers act as effective barriers in rheophilic fishes, it is likely that after the colonization of northern slopes of the Carpathians, *B. carpathicus* became “trapped” in this isolated, remote location, and was subjected to more extreme demographic fluctuations than its Central European, rheophilic congeners – *B. balcanicus* and *B. petenyi* [6], [9].Taking into account that LGM glaciers in the Carpathians had very narrow altitudinal range [42] and that the existence of Carpathian refugia were documented in a number of species (e.g. [43],[44]), it is possible that appropriate conditions for *B. carpathicus* could have existed in the central part of the Carpathian range during the entire period of glaciation. On the other hand, the end of the glacial period was accompanied by droughts that might have altered dramatically the functioning of riverine ecosystems, however, our data does not allow us to test those hypotheses. 

The current distribution of the Carpathian barbel can only be explained with an assumption that the species was able to pass segments of lowland rivers and cross watersheds several times. After the LGM, it is likely that *B. carpathicus* began to expand its range and crossed the Black Sea–Baltic watershed in a number of stream captures. Such a mode of dispersal of montane fishes has been documented in other parts of Europe in the case of the bullhead, *Cottus gobio* [45], [46] [47], and may be regarded as effective [31], [48], [49]. The range expansion probably comprised of several episodes after which the newly established population grew in number and served as a source of individuals for subsequent colonization [50]. Due to low genetic variation, a detailed picture of expansion cannot be unambiguously drawn, however, major patterns are well supported in our results. An assignment test performed in Structure and TESS show a few well defined clusters. Within the Danube basin the populations from the south-western part of the range (the Torysa, Hornad, Slana, Ipel, Morgó, Hron) consequently assign together independent of assumed *K* number. These populations also form a separate cluster in FCA analysis and are most likely derived from a single ancestral population. Populations from the central part of the Tisza basin do not assign consequently. There can be several explanations for this pattern. Firstly, this part of the range could have been colonized in several expansion episodes. Secondly, if the population was genetically diverse, a subsequent demographic bottleneck could cause diversification among descendant subpopulations. 

Our results indicate that expansion to the northern slopes of the Carpathians was accomplished in at least two or three independent episodes. Populations from the Mała Wisła, Strwiąż and Orava basin are genetically related and possess very low genetic variation, which suggest these three populations are remnants of the first expansion event to the northern slopes of the Carpathian range. Unfortunately, neither sequence genealogies in a long segment of mtDNA nor private allele distribution could shed light on the direction of expansion among those populations. Their genetic uniformity may suggest that either the founder population was genetically uniform or experienced a strong bottleneck right after crossing the watershed. The source of the colonizing individuals and the direction of expansion cannot be unambiguously identified. Variation within mitochondrial haplotypes greatly overlaps between the Vistula basin and south-western Slovakian populations of *B. carpathicus*, though both distance method (Table S1) and assignment tests using nuclear loci (Figures 4 and 5) show those populations as genetically distinct. The lowest *F*_*ST*_ values between those three populations and the populations from the Danube basin were found in comparison with populations from the Topl’a and Laborec rivers, which may suggest that this region was the source of the colonizing individuals. The second colonization event can be inferred from assignment tests for K=3 in Structure and TESS and includes populations from the Biała and Wisłok rivers, that again assign to populations from the Topl’a and Laborec rivers. The third wave of expansion took place between the Hornad and Dunajec basins (probably from the Hornad to the Poprad). Those populations are closely related while being different from the neighbouring populations. The *F*_*ST*_ values between them are some of the lowest observed among all pairwise comparisons (Table S1).

### Origin of genetic clines

When clines of genetic variation are observed along the possible postglacial expansion routes it is often assumed that they arise as a result of several consecutive bottlenecks [12], [51]. In our study, this pattern seems more complex. Clines of variation were observed both in the Danube and Vistula basins. In the Danube basin, indices of genetic variation decrease gradually westwards, while in the Vistula basin the highest variation was observed in the Dunajec basin and it declines both eastwards and westwards. The origins of the clines may, however, be very different. The cline of variation in the Danube basin (except for population from the Orava basin) can be attributed to expansion through watersheds or long stretches of lowland rivers. *Barbus carpathicus* is strongly rheophilic and longer lowland river segments form effective dispersal barriers [8], which is supported by high *F*_*ST*_ values between populations separated by river reaches with slow current (Table S1). Expansion through those barriers was probably accomplished by a limited number of individuals. Once the population is established in the appropriate habitat it grows in numbers until approaching the carrying capacity of the new site [50], which scenario is particularly likely in a cluster of populations from the south-western part of the Carpathian barbel range, that probably originated from a single population as evidenced by an assignment test in Structure but shows declining levels of variation from east to west. This result shows that clines of genetic variation may occur not only over large geographical scales [12], [50] but also among local populations isolated by effective barriers to gene flow. In the Vistula, the scenario has most likely been more complex. The first colonization probably established large, but genetically highly uniform populations, which resulted in the genetic proximity of currently vicariant populations (the Mała Wisła, Strwiąż, Orava basins). In contrast, subsequent expansion from the Hornad to Poprad and then to all basin of the Dunajec and the Uszwica probably consisted of a large group of genetically more variable individuals. Populations from the Hornad and Dunajec basins are genetically related and posses a similar level of genetic variation. A westward cline (the Raba, Skawinka, Skawa, Soła, Mała Wisła) shows, however, no significant admixture from the Dunajec and Uszwica, while some genetic admixture from populations from the Wisłok/San and the Wisłoka/Biała groups is visible from Structure results (Figure 4). The origin of the eastward cline is more difficult to explain. The population from the Biała show traces of admixture with individuals from other tributaries of the Dunajec, but with a strong genetic fraction shared with the Wisłoka population in the results from both Structure and TESS programs. Individuals from the Wisłok and San assign consequently to a single population, genetically related to the first wave of expansion. It is possible that this cline was formed by independent events and should not be considered as a true single cline. Such a hypothesis contradicts the previous findings of Konopiński et al. [5], which explained the observed variation in a part of the Vistula basin by expansion associated with a loss of variation. It is possible that such ‘admixture’ clines also exist in other species and that only range-wide studies such as this one can elucidate the true origins of the observed patterns. 

### Demographic changes

The Bayesian Skyline Plot shows that the demographic expansion of *B. carpathicus* started approximately 2–6 kya, which falls well into the mid-Holocene (Fig. 6). Demographic expansion is also evidenced by other genetic tests (Table 3). It appears that genetic drift preceding the expansion has erased all the variation that accumulated in the species since it diverged from its congeners in the Miocene. Our results may suggest that after the early bottleneck, the species encountered other demographic fluctuations. The genetic variation carried by individuals from the third colonization wave (from the Hornad to Dunajec and Uszwica) strongly altered the genetic composition of populations within the Dunajec and Uszwica river systems. It is very likely that between the first and third colonization episodes, the Dunajec and its tributaries had held populations of the Carpathian barbel – the Dunajec basin is the largest river system among the Carpathian tributaries of the Vistula, and offers a number of sites with habitats suitable for *B. carpathicus* [8]. The Dunajec was the most likely colonization route between the Vistula and the Orava basins – the Carpathian watershed is very low between the Dunajec and the Czarna Orawa and passes the flat area of the Podhale and northern Orava regions, that is surrounded by the high ranges of the Tatra and the Beskid Żywiecki mountains. In fact, populations from the Dunajec take intermediate positions on FCA plot (Fig. 3) suggesting that the Dunajec is admixed with genes from the Hornad. If the earlier waves of expansion were genetically uniform, one could hypothesise that the successful expansion of genes carried by individuals from the Hornad population could be explained by some selection-driven processes such as heterozygote advantage or positive selection. A similar process may have shaped variation in the Biała population, which appears to have retained a significant fraction of distinct genetic variation originating from the second colonization wave. If no selective pressures were involved, the Dunajec and its tributaries (except for the Biała) should be unoccupied or contain small populations that were overwhelmed by individuals from the Hornad. Also population from Uszwica clusters unambiguously with populations from the Dunajec, Łososina, Poprad and at lower K's with populations from the south-eastern part of the species’ range. Both FCA plot and *F*_*ST*_ values indicate that this population is more genetically similar to the population from the Hornad, thus, it is tempting to hypothesise the Uszwica was recolonized immediately after the third colonization episode, when the colonizing population was less intermixed with the remnant, original populations from the Dunajec river system. 

Demographic breakdowns suggested by genetic data would imply that *B. carpathicus* has very strict ecological requirements and is sensitive to habitat alterations. The species is present only within the Carpathian range and did not successfully colonize any northern tributaries of the Vistula which possess similar physical characteristics in their upland reaches. Such special habitat requirements suggest that *B. carpathicus* can be particularly susceptible to the climate trend and increasing instability caused by global warming. Although Carpathian barbels are not of economic value they form an important fraction of ecosystems’ biomass and their extinction may affect the viability of other fish populations. 

## Supporting Information

Table S1 ***F*_*ST*_ values (below diagonal), their statistical support (above diagonal), *F*_*IS*_ values across diagonal.**
(DOC)

## References

[1] Hewitt G (2000) The genetic legacy of the Quaternary ice ages. Nature 405: 907–913. doi:10.1038/35016000. PubMed: 10879524. 10879524

[2] Janko K, Lecointre G, DeVries A, Couloux A, Cruaud C et al. (2007) Did glacial advances during the Pleistocene influence differently the demographic histories of benthic and pelagic Antarctic shelf fishes? – Inferences from intraspecific mitochondrial and nuclear DNA sequence diversity. BMC Evol Biol 7: 220. doi:10.1186/1471-2148-7-220. PubMed: 17997847. 17997847 PMC2222253

[3] Strona G, Galli P, Montano S, Seveso D, Fattorini S (2012) Global-Scale Relationships between Colonization Ability and Range Size in Marine and Freshwater. Fish - PLOS ONE 7: e49465. doi:10.1371/journal.pone.0049465. 23185338 PMC3504041

[4] Schmitt T (2007) Molecular biogeography of Europe: Pleistocene cycles and postglacial trends. Front Zool 4: 11. doi:10.1186/1742-9994-4-11. PubMed: 17439649. 17439649 PMC1868914

[5] Konopiński MK, Amirowicz A, Kukuła K (2007) Probable direction of the postglacial colonization of rivers on northern slopes of the Carpathian Ridge by Barbus carpathicus (Teleostei: Cyprinidae) evidenced by cline of genetic variation. J Fish Biol 70: 406–415. doi:10.1111/j.1095-8649.2007.01479.x.

[6] Kotlík P, Berrebi P (2002) Genetic subdivision and biogeography of the Danubian rheophilic barb Barbus petenyi inferred from phylogenetic analysis of mitochondrial DNA variation. Mol Phylogenet Evol 24: 10–18. doi:10.1016/S1055-7903(02)00264-6. PubMed: 12128024. 12128024

[7] Kotlík P, Tsigenopoulos CS, Rab P, Berrebi P (2002) Two new Barbus species from the Danube River basin, with redescription of B-petenyi (Teleostei : Cyprinidae). Folia Zool 51: 227–240.

[8] Rembiszewski M, Rolik H (1975) Cyclostomata et Pisces. Catalogus faunae Poloniae, XXXVIII Warszawa: PWN . p. 249

[9] Kottelat M, Freyhof J (2007) Handbook of Freshwater European Fishes. Berlin: Kottelat, Cornol, Switzerland, And Freyhof P 646.

[10] Bernatchez L, Wilson CC (1998) Comparative phylogeography of nearctic and palearctic fishes. Mol Ecol 7: 431–452. doi:10.1046/j.1365-294x.1998.00319.x.

[11] Wong BBM, Keogh JS, McGlashan DJ (2004) Current and historical patterns of drainage connectivity in eastern Australia inferred from population genetic structuring in a widespread freshwater fish Pseudomugil signifer (Pseudomugilidae). Mol Ecol 13: 391–401. doi:10.1046/j.1365-294X.2003.02085.x. PubMed: 14717894. 14717894

[12] Wofford JEB, Gresswell RE, Banks MA (2005) Influence of barriers to movement on within-watershed genetic variation of coastal cutthroat trout. Ecol Appl 15: 628–637. doi:10.1890/04-0095.

[13] Hasselman DJ, Ricard D, Bentzen P (2013) Genetic diversity and differentiation in a wide ranging anadromous fish, American shad (Alosa sapidissima), is correlated with latitude. Mol Ecol 22: 1558–1573. doi:10.1111/mec.12197. PubMed: 23379260. 23379260

[14] Xiao WH, Zhang YP, Liu HZ (2001) Molecular systematics of Xenocyprinae (Teleostei : Cyprinidae): Taxonomy, biogeography, and coevolution of a special group restricted in east. Asia - Mol Phylogenet Evol 18: 163–173. doi:10.1006/mpev.2000.0879. 11161753

[15] Machordom A, Doadrio I (2001) Evidence of a cenozoic Betic-Kabilian connection based on freshwater fish phylogeography (Luciobarbus, Cyprinidae). Mol Phylogenet Evol 18: 252–263. doi:10.1006/mpev.2000.0876. PubMed: 11161760. 11161760

[16] Werle E, Schneider C, Renner M, Völker M, Fiehn W (1994) Convenient Single-Step, One Tube Purification of Pcr Products for Direct Sequencing. Nucleic Acids Res 22: 4354–4355. doi:10.1093/nar/22.20.4354. PubMed: 7937169. 7937169 PMC331970

[17] Kotlík P, Marková S, Choleva L, Bogutskaya NG, Ekmekçi FG et al. (2008) Divergence with gene flow between Ponto-Caspian refugia in an anadromous cyprinid Rutilus frisii revealed by multiple gene phylogeography. Mol Ecol 17: 1076–1088. doi:10.1111/j.1365-294X.2007.03638.x. PubMed: 18261049. 18261049

[18] Hall T (1999) BioEdit: a user-friendly biological sequence alignment editor and analysis program for Windows 95/98/NT. Nucleic Acids Symp Ser 41: 95–98.

[19] Chenuil A, Galtier N, Berrebi P (1999) A test of the hypothesis of an autopolyploid vs. allopolyploid origin for a tetraploid lineage: application to the genus Barbus (Cyprinidae). Heredity 82: 373–380. doi:10.1038/sj.hdy.6884890. PubMed: 10383655. 10383655

[20] Tong J, Wang Z, Yu X, Wu Q, Chu KH (2002) Cross-species amplification in silver carp and bighead carp with microsatellite primers of common carp. Mol Ecol Notes 2: 245–247. doi:10.1046/j.1471-8278.2002.00214.x.

[21] Turner TF, Dowling TE, Broughton RE, Gold JR (2004) Variable microsatellite markers amplify across divergent lineages of cyprinid fishes (subfamily Leusicinae). Conserv Genet 5: 279–281. doi:10.1023/B:COGE.0000029998.11426.ab.

[22] Vyskočilová M, Šimková A, Martin J-F (2007) Isolation and characterization of microsatellites in Leuciscus cephalus (Cypriniformes, Cyprinidae) and cross-species amplification within the family Cyprinidae. Mol Ecol Notes 7: 1150–1154. doi:10.1111/j.1471-8286.2007.01813.x.

[23] Librado P, Rozas J (2009) DnaSP v5: a software for comprehensive analysis of DNA polymorphism data. Bioinformatics 25: 1451–1452. doi:10.1093/bioinformatics/btp187. PubMed: 19346325. 19346325

[24] Clement M, Posada D, Crandall KA (2000) TCS: a computer program to estimate gene genealogies. Mol Ecol 9: 1657–1659. doi:10.1046/j.1365-294x.2000.01020.x. PubMed: 11050560. 11050560

[25] Dieringer D, Schlötterer C (2003) MICROSATELLITE ANALYSER (MSA): a platform independent analysis tool for large microsatellite data sets. Mol Ecol Notes 3: 167–169. doi:10.1046/j.1471-8286.2003.00351.x.

[26] Goudet J (1995) FSTAT (Version 1.2): A computer program to calculate F-statistics. J Hered 86: 485–486.

[27] Excoffier L, Lischer HEL (2010) Arlequin suite ver 3.5: a new series of programs to perform population genetics analyses under Linux and Windows. Mol Ecol Resour 10: 564–567. doi:10.1111/j.1755-0998.2010.02847.x. PubMed: 21565059. 21565059

[28] Slatkin M, Hudson RR (1991) Pairwise Comparisons of Mitochondrial-Dna Sequences in Stable and Exponentially Growing Populations. Genetics 129: 555–562. PubMed: 1743491. 1743491 10.1093/genetics/129.2.555 PMC1204643

[29] Excoffier L (2004) Patterns of DNA sequence diversity and genetic structure after a range expansion: lessons from the infinite-island model. Mol Ecol 13: 853–864. doi:10.1046/j.1365-294X.2003.02004.x. PubMed: 15012760. 15012760

[30] Drummond AJ, Suchard MA, Xie D, Rambaut A (2012) Bayesian Phylogenetics with BEAUti and the BEAST 1 7. Mol Biol Evol 29: 1969–1973 doi:10.1093/molbev/mss075. PMC3408070 22367748

[31] Burridge CP, Craw D, Fletcher D, Waters JM (2008) Geological dates and molecular rates: Fish DNA sheds light on time dependency. Mol Biol Evol 25: 624–633. doi:10.1093/molbev/msm271. PubMed: 18281273. 18281273

[32] Weir B, Cockerham C (1984) Estimating F-Statistics for the Analysis of Population-Structure. Evolution 38: 1358–1370. doi:10.2307/2408641. 28563791

[33] Belkhir K, Borsa P, Chikhi L, Raufaste N, Bonhomme F (1996) GENETIX 4 05, logiciel sous Windows TM pour la génétique des populations. Laboratoire Génome, Populations, Interactions, CNRS UMR 5171, Université de Montpellier II, Montpellier (France) Available: http://kimura.univ-montp2.fr/genetix/. Accessed 26 July 2013

[34] Pritchard JK, Stephens M, Donnelly P (2000) Inference of population structure using multilocus genotype data. Genetics 155: 945–959. PubMed: 10835412. 10835412 10.1093/genetics/155.2.945 PMC1461096

[35] Hubisz MJ, Falush D, Stephens M, Pritchard JK (2009) Inferring weak population structure with the assistance of sample group information. Mol Ecol Resour 9: 1322–1332. doi:10.1111/j.1755-0998.2009.02591.x. PubMed: 21564903. 21564903 PMC3518025

[36] Evanno G, Regnaut S, Goudet J (2005) Detecting the number of clusters of individuals using the software STRUCTURE: a simulation study. Mol Ecol 14: 2611–2620. doi:10.1111/j.1365-294X.2005.02553.x. PubMed: 15969739. 15969739

[37] Earl DA, vonHoldt BM (2012) STRUCTURE HARVESTER: a website and program for visualizing STRUCTURE output and implementing the Evanno method. Conserv Genet Resour 4: 359–361. doi:10.1007/s12686-011-9548-7.

[38] Jakobsson M, Rosenberg NA (2007) CLUMPP: a cluster matching and permutation program for dealing with label switching and multimodality in analysis of population structure. Bioinformatics 23: 1801–1806. doi:10.1093/bioinformatics/btm233. PubMed: 17485429. 17485429

[39] Chen C, Durand E, Forbes F, François O (2007) Bayesian clustering algorithms ascertaining spatial population structure: a new computer program and a comparison study. Mol Ecol Notes 7: 747–756. doi:10.1111/j.1471-8286.2007.01769.x.

[40] François O, Ancelet S, Guillot G (2006) Bayesian clustering using hidden Markov random fields in spatial population genetics. Genetics 174: 805–816. doi:10.1534/genetics.106.059923. PubMed: 16888334. 16888334 PMC1602073

[41] Saitoh K, Sado T, Mayden RL, Hanzawa N, Nakamura K et al. (2006) Mitogenomic evolution and interrelationships of the cypriniformes (Actinopterygii : Ostariophysi): The first evidence toward resolution of higher-level relationships of the world’s largest freshwater fish clade based on 59 whole mitogenome sequences. J Mol Evol 63: 826–841. doi:10.1007/s00239-005-0293-y. PubMed: 17086453. 17086453

[42] Urdea P, Reuther A (2009) Some new data concerning the Quaternary glaciation in the Romanian Carpathians. Geogr Pannonica 13: 41–52.

[43] Kotlík P, Deffontaine V, Mascheretti S, Zima J, Michaux JR et al. (2006) A northern glacial refugium for bank voles (Clethrionomys glareolus). Proc Natl Acad Sci U S A 103: 14860–14864. doi:10.1073/pnas.0603237103. PubMed: 17001012. 17001012 PMC1595441

[44] Schmitt T, Varga Z (2012) Extra-Mediterranean refugia: The rule and not the exception? Front Zool 9. doi:10.1186/1742-9994-9-22. PMC3462695 22953783

[45] Riffel M, Schreiber A (1995) Coarse-grained population structure in Central European sculpin (Cottus gobio L): Secondary contact or ongoing genetic drift? J Zool Syst Evol Res 33: 173–184.

[46] Slechtová V, Bohlen J, Freyhof J, Persat H, Delmastro GB (2004) The Alps as barrier to dispersal in cold-adapted freshwater fishes? Phylogeographic history and taxonomic status of the bullhead in the Adriatic freshwater drainage. Mol Phylogenet Evol 33: 225–239. doi:10.1016/j.ympev.2004.05.005. PubMed: 15324851. 15324851

[47] Hänfling B, Brandl R (1998) Genetic differentiation of the bullhead Cottus gobio L. across watersheds in Central Europe: Evidence for two taxa. Heredity 80: 110–117. doi:10.1046/j.1365-2540.1998.00279.x.

[48] Hocutt C (1979) Drainage Evolution and Fish Dispersal in the Central Appalachians - Summary. Geol Soc Am Bull 90: 129–130. Available online at: doi:10.1130/0016-7606(1979)90<129:DEAFDI>2.0.CO;2

[49] Houston DD, Shiozawa DK, Riddle BR (2010) Phylogenetic relationships of the western North American cyprinid genus Richardsonius, with an overview of phylogeographic structure. Mol Phylogenet Evol 55: 259–273. doi:10.1016/j.ympev.2009.10.017. PubMed: 19874904. 19874904

[50] Taberlet P, Fumagalli L, Wust-Saucy AG, Cosson JF (1998) Comparative phylogeography and postglacial colonization routes in Europe. Mol Ecol 7: 453–464. doi:10.1046/j.1365-294x.1998.00289.x. PubMed: 9628000. 9628000

[51] Excoffier L, Ray N (2008) Surfing during population expansions promotes genetic revolutions and structuration. Trends Ecol Evol 23: 347–351. doi:10.1016/j.tree.2008.04.004. PubMed: 18502536. 18502536

