# Supplementary material for: Back from the Brink: The Holocene History of the Carpathian Barbel Barbus carpathicus
Source: PLoS One. 2013 Dec 12;8(12):e82464. doi: 10.1371/journal.pone.0082464 (PMC3861402; doi:10.1371/journal.pone.0082464)
Supplement: Table S1 — FST values (below diagonal), their statistical support (above diagonal), FIS values across diagonal. (DOC) [file pone.0082464.s001.doc]

Table S1. *F_ST_*  values (below diagonal), their statistical support (above diagonal), *F_IS_* values across diagonal.

|  | Somes | Iza | Uh | Laborec | Topl’a | Torysa | Hornad | Slana | Morgó | Ipel | Hron | Orava | Mała Wisła | Soła | Skawa | Skawinka | Raba | Uszwica | Łososina | Dunajec | Poprad | Biała | Wisłoka | Wisłok | Lower San | Upper San | Strwiąż |
| --- | --- | --- | --- | --- | --- | --- | --- | --- | --- | --- | --- | --- | --- | --- | --- | --- | --- | --- | --- | --- | --- | --- | --- | --- | --- | --- | --- |
| Somes | **0.090** | *0.149* | *3*10^-4^* | 10^-4^ | 10^-4^ | 10^-4^ | *2*10^-4^* | 10^-4^ | 10^-4^ | *0.006* | 10^-4^ | 10^-4^ | 10^-4^ | 10^-4^ | 10^-4^ | 10^-4^ | 10^-4^ | *0.000* | 10^-4^ | 10^-4^ | 10^-4^ | 10^-4^ | 10^-4^ | 10^-4^ | 10^-4^ | 10^-4^ | 10^-4^ |
| Iza | *0.013* | **0.121** | *3*10^-4^* | 10^-4^ | 10^-4^ | 10^-4^ | *2*10^-4^* | 10^-4^ | *0.001* | *0.025* | 10^-4^ | 10^-4^ | 10^-4^ | 10^-4^ | 10^-4^ | 10^-4^ | 10^-4^ | 10^-4^ | 10^-4^ | 10^-4^ | 10^-4^ | 10^-4^ | 10^-4^ | 10^-4^ | 10^-4^ | 10^-4^ | 10^-4^ |
| Uh | *0.049* | *0.045* | **0.048** | 10^-4^ | 10^-4^ | 10^-4^ | 10^-4^ | 10^-4^ | 10^-4^ | *0.003* | 10^-4^ | 10^-4^ | 10^-4^ | 10^-4^ | 10^-4^ | 10^-4^ | 10^-4^ | 10^-4^ | 10^-4^ | 10^-4^ | 10^-4^ | 10^-4^ | 10^-4^ | 10^-4^ | 10^-4^ | 10^-4^ | 10^-4^ |
| Laborec | 0.112 | 0.095 | 0.050 | **0.032** | 10^-4^ | 10^-4^ | 10^-4^ | 10^-4^ | 10^-4^ | 10^-4^ | 10^-4^ | 10^-4^ | 10^-4^ | 10^-4^ | 10^-4^ | 10^-4^ | 10^-4^ | 10^-4^ | 10^-4^ | 10^-4^ | 10^-4^ | 10^-4^ | 10^-4^ | 10^-4^ | 10^-4^ | 10^-4^ | 10^-4^ |
| Topl’a | 0.109 | 0.108 | 0.078 | 0.044 | **0.082** | 10^-4^ | 10^-4^ | 10^-4^ | 10^-4^ | 10^-4^ | 10^-4^ | 10^-4^ | 10^-4^ | 10^-4^ | 10^-4^ | 10^-4^ | 10^-4^ | 10^-4^ | 10^-4^ | 10^-4^ | 10^-4^ | 10^-4^ | 10^-4^ | 10^-4^ | 10^-4^ | 10^-4^ | 10^-4^ |
| Torysa | 0.070 | 0.063 | 0.092 | 0.167 | 0.195 | **0.027** | *0.003* | *0.002* | *2*10^-4^* | *0.185* | 10^-4^ | 10^-4^ | 10^-4^ | 10^-4^ | 10^-4^ | 10^-4^ | 10^-4^ | 10^-4^ | 10^-4^ | 10^-4^ | 10^-4^ | 10^-4^ | 10^-4^ | 10^-4^ | 10^-4^ | 10^-4^ | 10^-4^ |
| Hornad | *0.054* | *0.054* | 0.106 | 0.148 | 0.168 | *0.026* | **0.037** | *0.001* | 10^-4^ | *0.015* | 10^-4^ | 10^-4^ | 10^-4^ | 10^-4^ | 10^-4^ | 10^-4^ | 10^-4^ | 10^-4^ | 10^-4^ | 10^-4^ | 10^-4^ | 10^-4^ | 10^-4^ | 10^-4^ | 10^-4^ | 10^-4^ | 10^-4^ |
| Slana | 0.074 | 0.094 | 0.134 | 0.200 | 0.232 | *0.030* | *0.047* | **0.067** | *0.009* | *0.099* | 10^-4^ | 10^-4^ | 10^-4^ | 10^-4^ | 10^-4^ | 10^-4^ | 10^-4^ | 10^-4^ | 10^-4^ | 10^-4^ | 10^-4^ | 10^-4^ | 10^-4^ | 10^-4^ | 10^-4^ | 10^-4^ | 10^-4^ |
| Morgó | 0.109 | 0.096 | 0.153 | 0.253 | 0.299 | *0.096* | 0.147 | *0.071* | **0.104** | *0.072* | *0.036* | 10^-4^ | 10^-4^ | 10^-4^ | 10^-4^ | 10^-4^ | 10^-4^ | 10^-4^ | 10^-4^ | 10^-4^ | 10^-4^ | 10^-4^ | 10^-4^ | 10^-4^ | 10^-4^ | 10^-4^ | 10^-4^ |
| Ipel | *0.131* | *0.090* | *0.126* | 0.218 | 0.284 | *0.034* | *0.102* | *0.062* | *0.102* | **-0.167** | *0.031* | 10^-4^ | 10^-4^ | 10^-4^ | 10^-4^ | 10^-4^ | 10^-4^ | 10^-4^ | *0.006* | *0.003* | *0.013* | 10^-4^ | 10^-4^ | 10^-4^ | 10^-4^ | 10^-4^ | 10^-4^ |
| Hron | 0.162 | 0.176 | 0.232 | 0.312 | 0.344 | 0.120 | 0.139 | 0.081 | *0.067* | *0.130* | **0.406*** | 10^-4^ | 10^-4^ | 10^-4^ | 10^-4^ | 10^-4^ | 10^-4^ | 10^-4^ | 10^-4^ | 10^-4^ | 10^-4^ | 10^-4^ | 10^-4^ | 10^-4^ | 10^-4^ | 10^-4^ | 10^-4^ |
| Orava | 0.255 | 0.287 | 0.200 | 0.155 | 0.151 | 0.296 | 0.284 | 0.350 | 0.481 | 0.473 | 0.491 | **-0.066** | 10^-4^ | 10^-4^ | 10^-4^ | 10^-4^ | 10^-4^ | 10^-4^ | 10^-4^ | 10^-4^ | 10^-4^ | 10^-4^ | 10^-4^ | 10^-4^ | 10^-4^ | 10^-4^ | 10^-4^ |
| Mała Wisła | 0.232 | 0.291 | 0.237 | 0.185 | 0.132 | 0.288 | 0.265 | 0.340 | 0.485 | 0.480 | 0.476 | 0.101 | **0.036** | 10^-4^ | *0.075* | *0.005* | 10^-4^ | 10^-4^ | 10^-4^ | 10^-4^ | 10^-4^ | 10^-4^ | 10^-4^ | 10^-4^ | *2*10^-4^* | 10^-4^ | 10^-4^ |
| Soła | 0.205 | 0.274 | 0.197 | 0.162 | 0.146 | 0.266 | 0.247 | 0.287 | 0.426 | 0.402 | 0.419 | 0.116 | 0.062 | **-0.012** | 10^-4^ | *2*10^-4^* | 10^-4^ | 10^-4^ | 10^-4^ | 10^-4^ | 10^-4^ | 10^-4^ | 10^-4^ | 10^-4^ | 10^-4^ | 10^-4^ | 10^-4^ |
| Skawa | 0.172 | 0.231 | 0.180 | 0.140 | 0.090 | 0.259 | 0.227 | 0.300 | 0.424 | 0.417 | 0.430 | 0.087 | *0.013* | 0.048 | **-0.036** | *0.149* | *0.003* | 10^-4^ | 10^-4^ | 10^-4^ | 10^-4^ | 10^-4^ | 10^-4^ | 10^-4^ | 10^-4^ | *2*10^-4^* | 10^-4^ |
| Skawinka | 0.175 | 0.218 | 0.168 | 0.120 | 0.084 | 0.242 | 0.223 | 0.288 | 0.410 | 0.380 | 0.420 | 0.110 | *0.025* | *0.040* | *0.008* | **0.003** | *0.109* | 10^-4^ | 10^-4^ | 10^-4^ | 10^-4^ | 10^-4^ | 10^-4^ | 10^-4^ | *0.001* | *0.000* | 10^-4^ |
| Raba | 0.166 | 0.205 | 0.170 | 0.126 | 0.098 | 0.226 | 0.204 | 0.265 | 0.399 | 0.348 | 0.404 | 0.131 | 0.053 | 0.049 | *0.027* | *0.008* | **-0.076** | 10^-4^ | 10^-4^ | 10^-4^ | 10^-4^ | 10^-4^ | 10^-4^ | 10^-4^ | 10^-4^ | 10^-4^ | 10^-4^ |
| Uszwica | *0.078* | 0.137 | 0.111 | 0.176 | 0.198 | 0.121 | 0.105 | 0.126 | 0.213 | 0.238 | 0.236 | 0.303 | 0.304 | 0.229 | 0.235 | 0.252 | 0.243 | **0.040** | 10^-4^ | 10^-4^ | 10^-4^ | 10^-4^ | 10^-4^ | 10^-4^ | 10^-4^ | 10^-4^ | 10^-4^ |
| Łososina | 0.063 | 0.078 | 0.055 | 0.072 | 0.090 | 0.073 | 0.064 | 0.087 | 0.195 | *0.121* | 0.229 | 0.149 | 0.142 | 0.103 | 0.110 | 0.092 | 0.081 | 0.095 | **-0.059** | *0.710* | *0.495* | 10^-4^ | 10^-4^ | 10^-4^ | 10^-4^ | 10^-4^ | 10^-4^ |
| Dunajec | 0.077 | 0.109 | 0.076 | 0.092 | 0.119 | 0.088 | 0.075 | 0.090 | 0.230 | *0.158* | 0.256 | 0.179 | 0.169 | 0.099 | 0.122 | 0.108 | 0.085 | 0.098 | *-0.005* | **-0.007** | *0.105* | *2*10^-4^* | 10^-4^ | 10^-4^ | 10^-4^ | 10^-4^ | 10^-4^ |
| Poprad | 0.069 | 0.089 | 0.074 | 0.100 | 0.122 | 0.066 | 0.051 | 0.068 | 0.183 | *0.103* | 0.194 | 0.162 | 0.157 | 0.109 | 0.134 | 0.120 | 0.101 | 0.089 | *-0.001* | *0.007* | **0.048** | 10^-4^ | 10^-4^ | 10^-4^ | 10^-4^ | 10^-4^ | 10^-4^ |
| Biała | 0.112 | 0.157 | 0.123 | 0.108 | 0.103 | 0.173 | 0.146 | 0.160 | 0.273 | 0.251 | 0.290 | 0.142 | 0.126 | 0.052 | 0.092 | 0.083 | 0.070 | 0.129 | 0.045 | *0.030* | 0.052 | **0.069** | 10^-4^ | 10^-4^ | 10^-4^ | 10^-4^ | 10^-4^ |
| Wisłoka | 0.114 | 0.155 | 0.118 | 0.094 | 0.085 | 0.190 | 0.175 | 0.200 | 0.288 | 0.243 | 0.301 | 0.123 | 0.106 | 0.044 | 0.076 | 0.055 | 0.050 | 0.165 | 0.064 | 0.065 | 0.078 | 0.026 | **0.072** | 10^-4^ | 10^-4^ | 10^-4^ | 10^-4^ |
| Wisłok | 0.258 | 0.303 | 0.280 | 0.216 | 0.160 | 0.312 | 0.296 | 0.363 | 0.507 | 0.504 | 0.504 | 0.246 | 0.062 | 0.098 | 0.070 | 0.048 | 0.044 | 0.353 | 0.170 | 0.191 | 0.183 | 0.132 | 0.108 | **-0.005** | *0.002* | 10^-4^ | 10^-4^ |
| Lower San | 0.265 | 0.296 | 0.269 | 0.202 | 0.147 | 0.306 | 0.295 | 0.364 | 0.495 | 0.469 | 0.491 | 0.172 | *0.036* | 0.090 | 0.061 | *0.028* | 0.034 | 0.358 | 0.161 | 0.188 | 0.178 | 0.139 | 0.100 | *0.021* | **0.023** | *0.049* | 10^-4^ |
| Upper San | 0.276 | 0.300 | 0.267 | 0.194 | 0.138 | 0.311 | 0.300 | 0.366 | 0.497 | 0.467 | 0.500 | 0.157 | 0.043 | 0.093 | 0.059 | *0.040* | 0.034 | 0.357 | 0.159 | 0.183 | 0.178 | 0.130 | 0.101 | 0.033 | *0.007* | **-0.028** | 10^-4^ |
| Strwiąż | 0.427 | 0.470 | 0.386 | 0.291 | 0.255 | 0.426 | 0.415 | 0.516 | 0.692 | 0.717 | 0.660 | 0.175 | 0.087 | 0.197 | 0.146 | 0.187 | 0.207 | 0.484 | 0.288 | 0.343 | 0.277 | 0.240 | 0.225 | 0.218 | 0.169 | 0.156 | **0.011** |

*F_ST_*  values non-significant after Bonferroni correction in italic. Significant *F_IS_*  value indicated by asterisk
